# 

**Published:** 1908-07-04

**Authors:** 


THE MODERN MEDICAL JOURNAL.

Telephone Number: 2734 Gerrard.

NEW SERIES. No. 69, Vol. III. No. 1141. Vol. XLIV. — SATURDAY, JULY 4TH, 1908. — PRICE THREEPENCE.

